# Handwashing effect on diarrheal incidence in children under 5 years old in rural eastern Ethiopia: a cluster randomized controlled trial

**DOI:** 10.1186/s41182-021-00315-1

**Published:** 2021-03-23

**Authors:** Ephrem Tefera Solomon, Sirak Robele Gari, Helmut Kloos, Bezatu Mengistie Alemu

**Affiliations:** 1 Ethiopian Institute of Water Resources, Addis Ababa University, Addis Ababa, Ethiopia; 2 College of Health and Medical Sciences, Haramaya University, Harar, Ethiopia; 3 San Francisco Medical Center, University of California, San Francisco, CA USA

**Keywords:** Handwashing compliance, Under-five children, Childhood diarrhea, Ethiopia

## Abstract

**Background:**

Handwashing with soap reduces diarrheal diseases burden considerably. However, the importance of handwashing in homes has received little attention in rural eastern Ethiopia. The effectiveness of handwashing may be reduced by lack of information on when and in what event hands must be washed, the frequency of handwashing, the individual who should wash his/her hands, and the procedure of handwashing. In these areas, indicators of adherence to handwashing are yet to be established. This study aimed at assessing the efficiency of handwashing on reducing diarrheal disease in children under 5 years old in rural *kebeles* of Dire Dawa, east Ethiopia.

**Methods:**

Community-based cluster randomized controlled trial was conducted in rural *kebeles* of Dire Dawa for 4 months starting from October 2018 to January 2019. Selected clusters were randomized in intervention and control arms using draw method and data collectors conducted the baseline survey. Households assigned to the intervention group were given two bars of plain soap on a bi-monthly basis together with information promoting hand hygiene. Control households were allowed to continue their habitual handwashing practices. We compared the diarrheal incidences of the intervention and non-intervention households. Generalized estimation equations using Poisson family and log choice of the link was employed to calculate adjusted incidence rate ratio with its 95% confidence interval.

**Results:**

We recorded a significant lesser diarrheal incidence in the handwashing arm than in the non-intervention arm (6.9 versus 13.8 episodes per 100 person weeks of observation). In all, there was a 41% reduction in diarrheal incidence in the intervention arm in relation to the non-intervention arm.

**Conclusion:**

Handwashing with soap complemented with hand hygiene promotion significantly decreased diarrheal episodes in children under 5 years old in rural *kebeles* of Dire Dawa. We recommend the promotion and adaptation of washing hands using soap at recommended times to be an effective means of reducing childhood diarrhea morbidity in rural populations of Ethiopia towards achieving the Sustainable Development Goal 6.

**Trial registration:**

PACTR, PACTR201807815961394. Registered 16 July 2018,

**Supplementary Information:**

The online version contains supplementary material available at 10.1186/s41182-021-00315-1.

## Background

Diarrhea is a severe worldwide health problem, predominantly in LMICs (low-income and middle-income countries) [1]. It was a primary killer of children, responsible for nearly 8% of the entire deaths in children under 5 years old worldwide in the year 2017 [2]. The United Nations set out 17 SDG (Sustainable Development Goals), the sixth one of which aims to avail water and sanitation to all people in the world by 2030 [3].

Handwashing after contact with excreta is poorly practiced globally, despite the likely positive health benefits. The global mean prevalence of handwashing was estimated at 19% [4], and it is estimated that inadequate hand hygiene results in nearly 300,000 deaths annually, with the majority of deaths being among children younger than 5 years old [5]. Despite the fact that proper handwashing with soap can reduce the risk of diarrhea by 42–48% [6], diarrheal pathogens are transmitted by the fecal-oral route [7]. In Ethiopia, the prevalence of diarrhea among under-five children was 12.1% in 2016 [8]. Some of the risk factors of diarrheal morbidity in children under the age of 5 years in Ethiopia were as follows: sex of the child, the number of under-five children, household economic status, the presence of feces around the pit-hole, and absence of refuse disposal facilities [9, 10]. Therefore, improved handwashing methods play key role on reducing diarrheal incidence [11]. Specifically, handwashing after defecating and touching feces, before preparation of food and before intake of food may minimize diarrhea risk [1]. Washing hands with plain soap is effective in eliminating pathogenic microorganisms [12].

The diseases burden related to inadequate WASH are avertable with confirmed, cost-effective interventions [13]. A recently updated meta-analysis found large potential reductions in the risk of diarrheal disease through interventions aimed at improving in drinking water, sanitation, and hygiene [14]. Several studies reported that handwashing with soap produced between 25 and 53% reduction of diarrheal incidence and prevalence in children under 5 years old [11, 15–19]. Characteristics that were independently significantly associated with handwashing were being an adult caregiver, mother’s education above primary level, educational level of household heads, ethnicity of the household head, household wealth index, having water available at the place to wash hands after toileting, improved water sources, having soap available at the place to wash hands after toileting, and access to improved sanitation facilities [20–22].

Interventions that improve the obtainability of water with soap at a designated handwashing places are expected to improve handwashing behavior [23] and thus generate broad public health benefits. However, changing handwashing habits is often difficult. In eastern Ethiopia, Jigjiga District, an interventional study on the effect of handwashing on diarrhea of under-fives was carried out by Hashi and colleagues on 1224 under-five children with measurement of compliance with the intervention using drinking water quality test [19]. However, in our study, compliance was measured using surrogate measure (soap wrappers collection) in addition to water quality test. Additionally, in the study area of rural eastern Ethiopia, the importance of handwashing in homes has received little attention, and specific aspects of handwashing such as in what event and when hands must be washed, the frequency of handwashing, the individual who should wash his/her hands, and the procedure of handwashing, are yet to be addressed. Furthermore, indicators of adherence to handwashing are yet to be established. This study aimed at assessing the efficiency of handwashing on reducing diarrheal disease in children under 5 years old in rural *kebeles* of Dire Dawa, east Ethiopia.

## Methods

### Study area and period

Dire Dawa Administration is located in the eastern Ethiopia at about 500 km east of Addis Ababa. This small region is administratively divided into nine urban and 38 rural peasant associations in four operational *wereda* (*Wereda* are the third-level administrative division of Ethiopia below Region and Zone). As per the report by Water, Mine and Energy Bureau of Dire Dawa, potable water in the rural *kebeles* were provided by wells and springs. Health services are being delivered to the rural *kebeles* by seven health centers and 33 health posts (Dire Dawa administration Regional Health Bureau: 2017 six months report (unpublished)). This cluster randomized controlled trial was conducted in rural *kebeles* of Dire Dawa from October 2018 to January 2019.

### Inclusion criteria

In our study, the included households need to have a minimum of one child under 5 years old and the child should not suffer from chronic illness.

### Exclusion criteria

The excluded households were as follows: those households with mothers/caregivers who were incapable of responding to the questionnaire due to severe illness and households with children under 5 years old who were suffering from persistent diarrhea (three and more unformed stools in a day persisting for greater than 14 days).

### Baseline survey and follow-up

The baseline survey was conducted using a structured and pre-tested questionnaire addressing pertinent behavioral, environmental, and socio-demographic factors as well as diarrheal prevalence in a couple of weeks prior to the survey. Afaan Oromo, the local language, was used for the interview. The questionnaire was administered by trained data collectors to mothers or caregivers of under-five children. After completing the baseline survey, data on the occurrence of diarrhea were collected [24] once every 2 weeks by the data collectors. A total of eight rounds of diarrheal occurrences were recorded using pre-structured follow-up visit forms.

### Procedure and design of the study

**Fig. 1:**
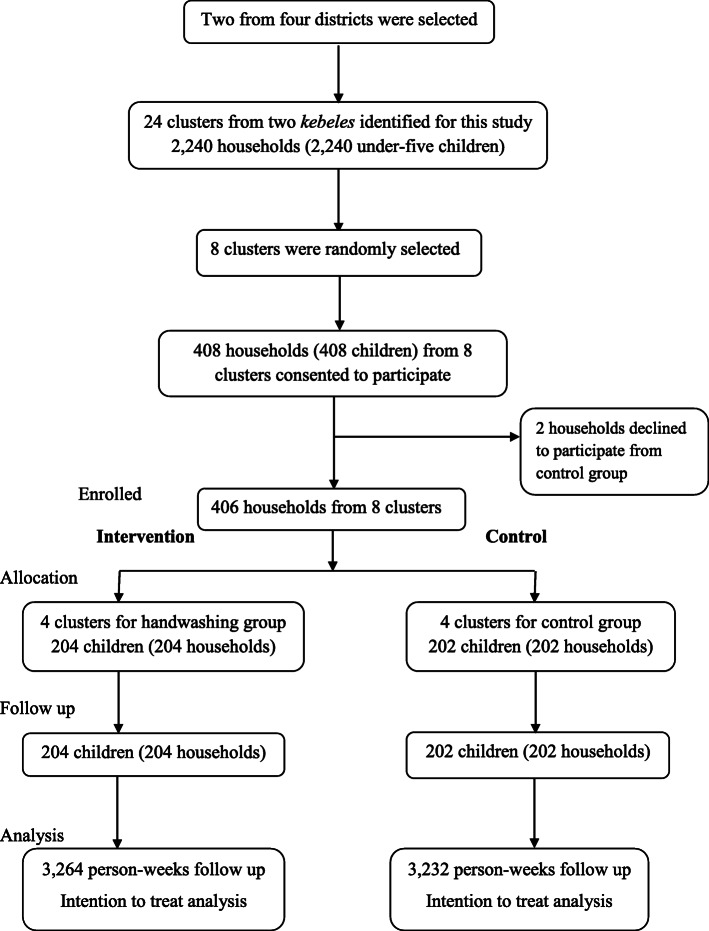
Selection and follow-up flow of study participants for the community randomized controlled trial, rural Dire Dawa, eastern Ethiopia, 2019

A cluster randomized controlled trial was carried out to assess the efficiency of handwashing of mothers/caregivers on reducing diarrheal diseases in children under 5 years old in rural *kebeles* of Dire Dawa from October 2018 to January 2019. The trial was begun the first week of October 2018 and completed in the last week of January 2019. Our rationale for randomizing at the cluster level other than the participant level was to avoid information contamination, so that individuals within a cluster are probably parallel and show comparable results [25]. The rural *kebeles* of Dire Dawa have four operational *wereda* or districts having 38 *kebeles* in total. Administratively, *kebele* will be divided to sub-*kebeles*. We randomly selected two districts from the total of four. Then, out of 12 *kebeles* in the two districts, two *kebeles* were selected based on their location which was five kilometers far apart in order to avoid information contamination. Twenty four clusters were found in the two *kebeles* out of which eight clusters were randomly selected for the trial. Four clusters were intervention arm and other four clusters the control arm (Fig. 1).

Diarrheal incidence was calculated as the ratio of counts of new cases of diarrhea by person weeks of observation [26]. We undertook eight series of interviews once every 2 weeks to determine the number of episodes of diarrhea for 16 weeks.

The principal investigator, in collaboration with the study team, arranged gathering with leaders of the community to allocate the clusters in intervention group (IG) and control group (CG). A unique identifier was given to each cluster and papers with identifiers were placed in a pot after folding them. Two collections of pieces of papers equal in number, labeled “IG” and “CG” were folded and placed in another pot. Two anonymous persons from the gathering who had not participated in labeling took one paper from the two pots in the presence of the community leaders and the two papers were paired. This process continued until all papers were paired. Unique identifiers paired with “IG” were allocated to the intervention arm and the others paired with “CG” were allocated to control arm.

Randomization of participants at cluster level is frequently promoted to reduce “contamination” of treatment among intervention and control arms [27]. After getting informed and written consent from every household, data collectors administered a baseline survey to the participants. Lastly, two bars of plain soap were distributed bi-monthly to all intervention households until the end of the study. In each 2-week round, soap was distributed on the first day of the first week starting on Monday, 8 October 2018 (Fig. 1).

### Sample size for under-five children and sample size determination for clusters

The sample size was calculated by taking 35% reduction in incidence of diarrhea [19] in the intervention arm when compared with the control arm. The following parameters were taken into consideration: 80% of power, 5% of significance level, 95% of confidence level, 10% for the possibility of refusal by the participants, and design effect with four folds as a result of clustering. These considerations generated 204 children in each arm and a total of 408 children for the study. The required number of clusters was calculated by the method established by Hayes and Bennett [28]. Accordingly, the intervention arm used four clusters and the control arm used four clusters.

### Study variables

The dependent variable was the occurrence of childhood diarrhea every 2 weeks. Child sex and age, number of under-five children per household, history of breastfeeding, family size, mother/caregiver age, availability of plain soap in the home, handwashing before food preparation, before intake of food, before feeding under-five children, after use of toilet, after contact with child feces, presence of refuse disposal facility, drinking water storage container, presence of watch in the house, presence of television in the house, mother’s education, availability of latrine, the household water source, time taken to fetch water for a round trip, and microbial water quality were the independent variables.

### Intervention

Mothers/caregivers of the intervention households were provided with two bars of plain soap. Information about hand hygiene for washing hands in the following five recommended times: before food preparation, before food intake, before feeding under-five child, after toilet use, and after cleaning the child’s bottom was given. Participants were instructed to keep the soap wrappers. The intervention providers collected the soap wrappers on a bi-monthly basis before giving each participant the next two bars of soap; the wrappers served as a measure of the study population’s adherence to the intervention. In each household, the intervention providers delivered a lesson on handwashing and demonstrated hand hygiene following recommendations of CDC [29]. Intervention providers received training on hand hygiene from the principal investigator. The guidelines are as follows: wet hands with water, lather hands completely with plain soap, rub hands for one third of a minute, wash using water, and dry [29].

Every time the intervention providers visited the intervention households, they reminded the mother/caregiver of hand hygiene once every 2 weeks. Moreover, the intervention providers were in close contact with the principal investigator, who reminded them of soap delivery. Mothers and caregivers in the intervention households including under-five children who were old enough to obey orders were encouraged by the intervention providers to practice handwashing at five critical times. The intervention providers did not encourage water treatment and other protective actions that can possibly lessen the occurrence of diarrhea. Handwashing at critical times was the only intervention mentioned.

Control households were allowed to continue their habitual handwashing practices. They were neither encouraged nor discouraged to practice handwashing with soap and water. However, data collectors visited each household in the control group on a bi-monthly basis to record occurrence of diarrhea in children under 5 years old.

### Operational definition of terms

Caregiver: Someone who takes care of an under-five child in the absence of the biological mother.

Control arm: Clusters that were not given plain soap and were allowed continuing their habitual handwashing practices.

Diarrhea: More frequent passage of loose stools than is normal for the individual, which is three times and more within 24 h [24].

Effect: The association of washing hands with plain soap at recommended times and the diarrheal incidence in children under 5 years old.

Family size: Total number of individuals living in a given household at the time of the survey.

Hand hygiene promotion: Educating mothers, guardians, and caregivers to wash their own hands and their children under 5 years old with plain soap at recommended times.

Handwashing group: Clusters supplied with plain soap for washing hands with soap at recommended times.

Handwashing: Involves the five steps of handwashing: wet with water; lather completely with soap, scrub the hands for one third of a minute, wash using water, and dry [29].

Household head: Mother or father who generates income for the family’s living expenses.

Improved water: Water gotten from public tap, pipe, protected well, protected spring, or rainwater collection

*Kebele*: Is the smallest administrative unit of Ethiopia.

Persistent diarrhea: The abrupt onset of three and more unformed stools in a day persisting for greater than 14 days [30].

Plain soap: A soap containing no triclocarban (1.2%) as an antibacterial agent.

Under-five children: Children of age range 6 to 59 months during the survey period.

Unimproved water: Water gotten from unprotected springs, unprotected dug wells, rivers, or streams.

### Data collection

At baseline, data were collected through a structured questionnaire. The questionnaire was first written in English, translated to Oromiffa, and back translated to English to ensure reliability of the Oromiffa translation. Data collection was carried out using the Oromiffa version by data collectors, intervention providers, and supervisors. The data collectors and intervention providers were grade 10 completed students wherein the supervisors were high school graduates. The field workers were recruited from the local areas and trained on proper data collection by the principal investigator within 2 days. At the end of the training, the data collection tool was pilot tested on 5% of the sample size in a neighboring *kebele* which was not included in the study conducted later. Based on the results of the pilot testing, the data collection tool was improved.

In this study, the main outcome variable was childhood diarrhea. In each 2-week round, an episode of diarrhea was counted as a new if the under-five child did not pass loose stools for 3 days [31]. The data collectors asked the mothers/caregivers when the diarrhea started and when it ended during each round of data collection; they also informed the mothers/caregivers either to remember carefully the date or to take notes about the date. Information was gathered by the data collectors on occurrence of diarrhea, handwashing practices, and the collection of soap wrapper on a bi-monthly basis for 16 weeks during the eight rounds.

The secondary outcome variable of this trial was compliance with the intervention by participating households. Compliance was measured by the number of soap wrappers collected; this measure was used as an indicator of soap use. The intervention households were also told to use the soap for bathing and washing dishes as well as washing clothes, because they were given 200gm bar of soap per week (two bars of soap for each 2-week round).

Random water samples were taken from 10% households of the intervention and control arms at baseline and end line of the study for microbiological water quality test. A 1% sodium thiosulfate was added in water samples to neutralize chlorine. The collected water samples kept in ice box were brought to the laboratory of Water Supply and Sewerage Authority within 4 h of being collected. The collected water samples were processed and examined by the membrane filtration technique to detect and quantify *Escherichia coli*, and this was performed by the Laboratory Technician in Dire Dawa Water Supply Sewerage Authority. Using the membrane filter technique, sample was passed through the membrane using a filter funnel and vacuum system. Any organisms in the sample are concentrated on the surface of the membrane. The membrane, with its trapped bacteria, is then placed in a special plate containing a pad saturated with lactose broth. The passage of nutrients through the filter during incubation facilitates the growth of organisms in the form of colonies, on the upper surface of the membrane that will be counted after 24 h of 44 °C incubation [32].

We tested the microbial quality of drinking water from both arms to assess indirectly any changes due to the intervention, especially for the intervention households. Continual handwashing decreases the chances of contamination of household- stored water and thus the risk of diarrhea. *E. coli* was considered as the reliable of all indicators of fecal contamination [33].

### Data analysis

Data were analyzed with STATA 15.0 after importing them from EPI-Data 3.1. Diarrheal incidence was compared between intervention and control arms by intention-to-treat analysis. Data of the intervention and non-intervention arms were paralleled at the baseline and during the follow-up period. Crude and adjusted incidence rate ratio with the accompanying 95% confidence intervals were calculated by generalized estimation equations (GEE) with Poisson family and log choice of the link after adjusting for potential confounders [34].

We used GEE instead of zero-inflated Poisson regression just for modeling longitudinal data. The GEE method is usually employed when the interest is to see the effect of population-averaged of factors on the outcomes of interest [35]. When the zero counts are in excess of the other counts, zero-inflated Poisson regression will be recommended. In our dataset, diarrhea counts are expressed in terms of presence or absence; that is why we did not use the zero-inflated model.

## Results

In the present study, 406 of the 408 (99.5%) selected households agreed to participate. Of these, 204 households in interventional arm and 202 households in non-intervention arm completed the entire 16 weeks of follow-up.

Median age of the mothers/caregivers was 28 (IQR 25–30) years and median age of children under 5 years old was 36 (IQR 24–48) months. The number of households per cluster was 51. Intervention and non-intervention arms were equivalent with regard to most of the measured variables (Table 1).

**Table 1:**
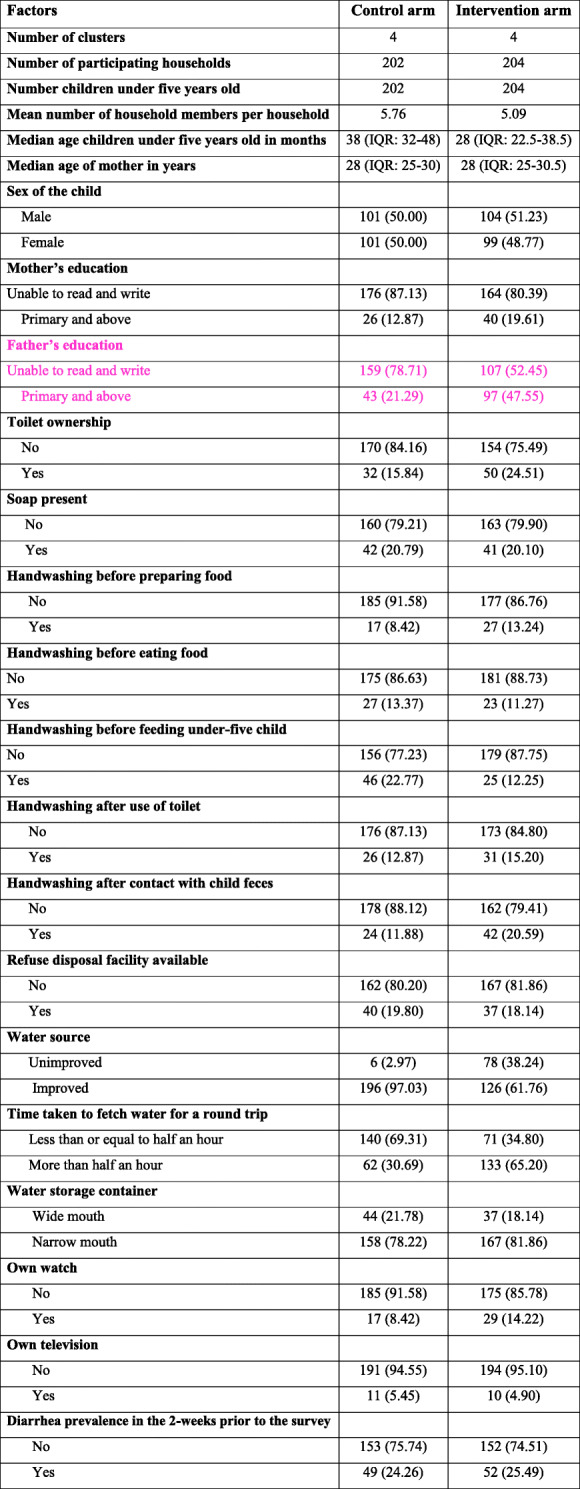
Socio-demographic, behavioral, and environmental variables and pre-intervention diarrhea of control and intervention groups at baseline, rural *kebeles* of Dire Dawa, 2019

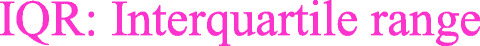

Intervention and non-intervention households had comparable features at the baseline; they were comparable in sex of the children, availability of plain soap in the home, and reported similar behavior of handwashing before preparing food, before eating food and after use of toilet. They also had similar numbers of refuse disposal facilities and similar household water storage containers and socio-economic status. The 2-week prevalence of diarrhea at the pre-intervention phase was 24.26% in those who were not covered by the intervention and 25.49% in those who were covered by the intervention (Table 1). The participants experienced no harm as a result of the intervention.

The prevalence of diarrhea at baseline was 24.9% whereas it was 17.7% at the end line of the study time thereby showing the effectiveness of the intervention. Similarly, microbial water quality test at baseline showed that 80.95% of the water samples tested was contaminated with *E. coli* at baseline; however, 47.62% was contaminated with *E. coli* at the end line of the study in the intervention households.

### Diarrheal incidence

**Fig. 2:**
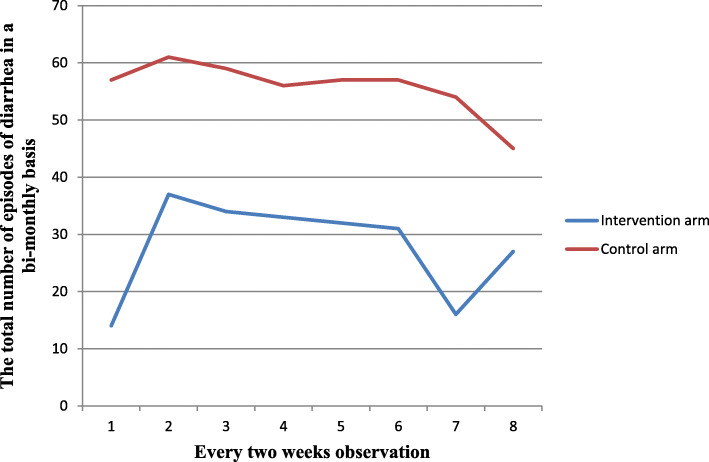
Total number of episodes of diarrhea recorded at 2-week observations for intervention and control groups in rural Dire Dawa, eastern Ethiopia, 2019

Four hundred forty-six diarrheal episodes were recorded in the control arm (13.8 episodes per 100 person weeks of observation), and 224 diarrheal episodes were recorded in the intervention arm (6.9 episodes per 100 person weeks of observation). Episode numbers of diarrhea on a bi-monthly basis against weeks of observation are shown in Fig. 2.

Having controlled for sex of the child, history of breastfeeding, number of household members, refuse disposal facility, availability of latrine, drinking water source, and household water storage container, reduced diarrhea risk was recorded in under-five children in the interventional arm ([adjusted incidence rate ratio] aIRR = 0.59, *CI* [95%] 0.53–0.65). An overall reduction of 41% in diarrheal incidence was observed in children under 5 years old in the intervention arm than those in the non-intervention arm (Table 2).

**Table 2:** Effect of handwashing on diarrheal incidence in children under 5 years old as analyzed by multivariable analysis in rural *kebeles* of Dire Dawa, east Ethiopia, 2019

Features	Crude IRR ***CI*** (95%)	Adjusted IRR ***CI*** (95%)	***p*** value
**Interventional arm**	0.59 (0.53–0.65)	0.59 (0.53–0.65)	< 0.001
Non-intervention arm	1	1	
**Age of the child**	1.00 (0.99–1.00)	1.00 (0.99–1.00)	0.991
**Sex of the child**			
Female	0.99 (0.92–1.08)	0.99 (0.92–1.08)	0.947
Male	1	1	
**History of breastfeeding**			
Yes	1.02 (0.81–1.27)	1.02 (0.81–1.27)	0.872
No	1	1	
**Number of household members**	1.00 (0.98–1.02)	1.00 (0.98–1.02)	0.807
**Refuse disposal facility present**			
Yes	1.01 (0.91–1.11)	1.01 (0.91–1.12)	0.907
No	1	1	
**Latrine present**			
Yes	0.99 (0.91–1.10)	0.99 (0.90–1.09)	0.856
No	1	1	
**Drinking water source for the household**			
Improved source	0.99 (0.91–1.08)	0.99 (0.91–1.07)	0.792
Unimproved source	1	1	
**Water storage container**			
Narrow necked	0.98 (0.89–1.09)	0.98 (0.88–1.09)	0.717
Wide necked	1	1	

*IRR* incidence rate ratio, *CI* confidence interval; the statistical test used is GEE

During the 16 weeks of observation, the diarrhea experience of under-five children in households of the interventional arm was 1% of 22,440 study days, wherein the diarrhea experience of the children in the non-intervention households was 2.01% of 22,220 study days (Table 3).

**Table 3:** Days gone with diarrhea against study arms in children under 5 year old, rural *kebeles* of Dire Dawa, east Ethiopia, 2019

Arms	No of diarrheal episodes	No of under-five children	Total days of observation	% of days with diarrhea
Handwashing arm	224	204	22,440	1.00
Control arm	446	202	22,220	2.01

*No* number

In the intervention, households’ handwashing practices exhibited different effects on reducing episodes of diarrheal disease among the various ranges of age in children under 5 years old. Higher effect (52%) was observed in children aged 1 to 2 years (Table 4).

**Table 4:** Intervention effect among diverse age range of children under 5 years old in rural *kebeles* of Dire Dawa, east Ethiopia, 2019

Age range	Control arm (***n*** = 202)			Interventional arm (***n*** = 204)			
	Counts of diarrheal episodes	PWO	Incidence of diarrhea	Counts of diarrheal episodes	PWO	Incidence of diarrhea	Reduction in %
**< 1 year**	36	240	15.0	47	608	7.7	48.67
**1 to 2 years**	180	1328	13.6	111	1712	6.5	52.21
**3 to 4 years**	230	1664	13.8	66	944	7.0	49.28

Incidence of diarrhea = counts of diarrheal episodes/100 person weeks of observation, PWO = person weeks of observation

### Household microbial water quality

At baseline, 80.95% and 80.0% of the sampled water from the intervention and control arms, respectively, were contaminated with *E. coli* and with no significant difference in counts (*p* = 0.311). After the intervention, significantly fewer samples from the intervention (47.62%) households than from the control households (85.0%) were contaminated (*p* = 0.033) (Table 5).

**Table 5:** Intervention and control households’ numbers with *E. coli* in drinking water at baseline and end line, in rural *kebeles* of Dire Dawa, east Ethiopia, 2019

***E. coli*** in drinking water	Intervention households ***N*** (%)	Control households ***N*** (%)	***p*** value
Base line	17 (80.95)	16 (80.00)	0.311
End of the study time	10 (47.62)	17 (85.00)	0.033

*N* sample size; significance level was set at *p* < 0.05

### Compliance with the intervention

Intervention providers collected 78.24% of the total number of soap wrappers distributed during the entire 16-week intervention.

With regard to drinking water treatment at baseline, 14 households (6.9%) in the intervention arm and 8 households (4.0%) in non-intervention arm treated the drinking water by various methods.

## Discussion

In this study, we assessed the efficiency of household handwashing on reduction of diarrheal diseases in children under 5 years old in rural *kebeles* of Dire Dawa. Handwashing with soap produced a 41.0% of reduction of diarrheal incidence in children under 5 years old in the interventional households than in the non-intervention households (aIRR = 0.59, *CI* [95%] 0.53–0.65). Additionally, for children who had episodes of diarrhea, the number of days with diarrhea in the intervention arm was half the number of days for those in the control arm.

The 41% reduction of diarrheal incidence of this study was lower than the 56% reduction reported in 2–5 year old children in a peri-urban slum of Dhaka City, Bangladesh, during a 1-year surveillance [36] and similar reductions in two trials in Karachi, Pakistan, 53% and 51%, respectively. It was also lower than the 48% reduction resulted from the conclusion of a systematic review [37]. The interventions in these studies were carried out for 1 year and 8 months, respectively, with visits once in a week [38, 39]. The lower reduction achieved in our study may be explained by the shorter study period (4 month) than in the above studies. The 41% reduction attained in the present study is consistent with results of similar studies in Kenya (41%) [18] and in Karachi, Pakistan (39%) [11], which also used plain soap. Our study results are also consistent with the conclusion of a recent systematic review that handwashing may decrease diarrheal disease risk by 40% [4] as well as with the conclusion of systematic review having a reduction in risk of 43% [40]. On the contrary, it is larger than trials in different countries such as Myanmar (30%) [15], Malawi (27%) [16], and India (25%) [17]. It is also larger than the conclusions of a meta-analysis (31%), systematic review (27%), and a recent updated meta-analysis and meta-regression 30% [14, 41, 42]. The fact that different critical times for handwashing were studied in the Myanmar trial may explain differences with our trial. Furthermore, the high reduction achieved in our study might be explained by greater compliance (78.24%).

The reduction in the incidence of diarrhea we achieved was by far greater than in studies carried out in a Calcutta slum in India [43] and in the Netherlands [44], both of which reported lack of intervention effectiveness. Moreover, the intervention and control households in the above studies were not comparable in most of their baseline characteristics, whereas they were fairly balanced in our study. These disparities may have contributed to these differences.

The quality of drinking water in water containers was improved after the handwashing intervention. This could be due to improved handwashing practices in the intervention households as clean hands protect the drinking water from being contaminated. This result corroborates a similar trial carried out in Jigjiga District in eastern Ethiopia [19], further indicating that regular handwashing prevents or reduces diarrhea transmission.

Handwashing with soap is not only effective in preventing diarrhea [38] but may also be even more effective in preventing diarrheal diseases than improving water supply and sanitation [45]. Unlike water treatment products, plain soap is easily accessible and affordable. The common price of a bar of soap in Ethiopian in retail shops is 10 Birr (0.31 US$). Furthermore, handwashing facilities are more manageable and can be constructed with local materials by households, unlike toilets.

This study has two limitations: first, we expected recall bias because we collected information about the occurrence of diarrhea once in a couple of weeks. Managing this limitation was by carefully training the data collectors to repeatedly ask the mothers/caregivers to specify the date when the diarrhea began and when it ended. Second, because the intervention material (bars of plain soap) was given to the participants free of charge, we anticipated that Hawthorne effect and courtesy bias might inflate the intervention effect. In contrast, these biases were decreased by using intervention providers independent of data collectors to distribute bars of soap to participants.

## Conclusion

This study reveals that, handwashing with soap complemented with hand hygiene promotion can significantly reduce diarrheal episodes in children under 5 years old in rural *kebeles* of Dire Dawa. Therefore, we recommend the promotion and adaptation of handwashing with soap at critical times as an effective, simple, and affordable means of reducing childhood diarrhea morbidity in rural populations of Ethiopia and other developing countries and thus for contributing to attainment of SDG 6 set by the United Nations.

## Supplementary Information

**Additional file 1.** Supplement file Questionnaire.

## Data Availability

The authors are willing to share the data on which results and conclusion are based upon request by the journal.

## Abbreviations

CG: Control group
GEE: Generalized estimation equations
IG: Intervention group
IQR: Interquartile range
