# Supplementary material for: Handwashing effect on diarrheal incidence in children under 5 years old in rural eastern Ethiopia: a cluster randomized controlled trial
Source: Trop Med Health. 2021 Mar 23;49:26. doi: 10.1186/s41182-021-00315-1 (PMC7989202; doi:10.1186/s41182-021-00315-1)
Supplement: Supplementary file 1 — Additional file 1. Supplement file Questionnaire. [file 41182_2021_315_MOESM1_ESM.docx]

# Appendix 4. Questionnaire

***To the interviewer, please encircle/write the answer!***

**4.1. Questions regarding background information**

| **No.** | **Questions** | **Options** | **Skip** | **Remark** |
| --- | --- | --- | --- | --- |
|  | Sex of the index under-five child | 1. Male 2. Female |  |  |
|  | **To the interviewer:ask the mother to bring you the birth certificate of the under-five child to write the age in months!**  What is the age of the index under-five child? | _________ in months |  |  |
|  | How many under-five children are found in the house? | 1. One 2. Two 3. Three and above |  |  |
|  | What is the age of the mother or caregiver or the guardian? | _______________ |  |  |
|  | What is the educational status of the mother or caregiver or the guardian? | 1. Illiterate 2. Grade 1 – 4 3. Grade 5 – 8 4. Grade 9 – 12 5. 12^+^ |  |  |
|  | What is the age of the father? | ______________ |  |  |
|  | What is the educational status of the father? | 1. Illiterate 2. Grade 1- 4 3. Grade 5 – 8 4. Grade 9 – 12 5. 12^+^ |  |  |
|  | What is the occupation of the father? | 1. Farmer 2. Merchant 3. Day laborer 4. Government employee |  |  |
|  | What is your monthly family income? | ______________ in Birr |  |  |
|  | Do you have latrine? | 1. No 2. Yes | **If the answer is no, skip to question # 112** | |
|  | **If the answer to question # 110 is yes**, how often you use the latrine? | 1. Sometimes 2. Always |  |  |
|  | **To the interviewer:** Do you observe feces around the pit-hole/on the slab? | 1. No 2. Yes |  |  |
|  | **To the interviewer:** Do you observe feces in and around the house? | 1. No 2. Yes |  |  |
|  | **To the interviewer:** Do you observe refuse in and around the house? | 1. No 2. Yes |  |  |
|  | **To the interviewer:** Do you see infestation of flies in the house? | 1. No 2. Yes |  |  |

**4.2. Questions regarding diarrhea**

| **No.** | **Questions** | **Options** | **Skip** | **Remark** |
| --- | --- | --- | --- | --- |
|  | Did your under-five child experienced diarrhea in the past 15 days? | 1. No 2. Yes |  |  |
|  | **To the interviewer:ask the mother to bring you the birth certificate of the under-five child!**  What was the birthweight in Kilogram OR in gram? | _______________ in Kg  OR           _______________ in gm |  |  |
|  | Did your under-five child practice open defecation? | 1. No 2. Yes |  |  |
|  | **To the interviewer: Please call the name of the child for this question!**  The last time (name) passed stools, what was done to dispose of the stools? | 1. Thrown in toilet 2. Thrown in garbage 3. Buried |  |  |
|  | Did you breast fed your under-five child? | 1. No 2. Yes |  |  |
|  | **Question to the mother directly:**  Did you experience diarrhea in the past 15 days? | 1. No 2. Yes |  |  |
|  | Do you have refuse disposal facility in your house? | 1. No 2. Yes |  |  |
|  | **To the interviewer don’t ask simply observe:**  From what the house floor is made? | 1. Dirt floor 2. Cement |  |  |

**4.3. Questions regarding household water**

| **No.** | **Questions** | **Options** | **Skip** | **Remark** |
| --- | --- | --- | --- | --- |
|  | What is your drinking water source? | 1. Piped water 2. Public tap 3. Rainwater collection 4. Unprotected dug well 5. Unprotected spring 6. River water (stream water) 7. Other (specify) _________ |  |  |
|  | How much it takes you to fetch water for a round trip? | 1. Less than half an hour 2. Half an hour 3. More than half an hour |  |  |
|  | About the household water storage container, is it? | 1. Wide-mouthed 2. Narrow-mouthed |  |  |
|  | What is your family size? | _____________ in number |  |  |

**4.4. Questions regarding handwashing**

| **No.** | **Questions** | **Options** | **Skip** | **Remark** |
| --- | --- | --- | --- | --- |
|  | **To the interviewer: ask the mother to show you the handwashing facility!**  Does the household have handwashing facility? | 1. No 2. Yes |  |  |
|  | Does the facility have soap? | 1. No 2. Yes |  | |
|  | **Question to the mother:** do you wash your hands before preparing food? | 1. No 2. Yes | **If the answer is no, skip to question # 132** | |
|  | **If the answer to the question # 130 is yes**, is it with soap? | 1. No 2. Yes |  |  |
|  | **Question to the mother:** do you wash your hands before eating food? | 1. No 2. Yes | **If the answer is no, skip to question # 134** | |
|  | **If the answer to the question # 132 is yes**, is it with soap? | 1. No 2. yes |  |  |
|  | **Question to the mother:** do you wash the hands of your under-five child before he/she eats? | 1. No 2. Yes | **If the answer is no, skip to question # 136** | |
|  | **If the answer to the question # 134 is yes**, is it with soap? | 1. No 2. Yes |  |  |
|  | **Question to the mother:** do you wash your hands before feeding your under-five child? | 1. No 2. Yes | **If the answer is no, skip to question # 138** | |
|  | **If the answer to the question # 136 is yes**, is it with soap? | 1. No 2. Yes |  |  |
|  | **Question to the mother:** do you wash your hands after use of toilet? | 1. No 2. Yes | **If the answer is no, skip to question # 140** | |
|  | **If the answer to the question # 138 is yes**, is it with soap? | 1. No 2. Yes |  |  |
|  | **Question to the mother:** do you wash the hands of your under-five child after he/she defecates? | 1. No 2. Yes | **If the answer is no, skip to question # 142** | |
|  | **If the answer to the question # 140 is yes**, is it with soap? | 1. No 2. Yes |  |  |
|  | **Question to the mother:** do you wash your hands after contact with child feces? | 1. No 2. Yes | **If the answer is no, skip to question # 144** | |
|  | **If the answer to the question # 142 is yes**, is it with soap? | 1. No 2. Yes |  |  |
|  | **Question to the mother:** do you wash your hands after eating food? | 1. No 2. Yes | **If the answer is no, skip to question # 146** | |
|  | **If the answer to the question # 144 is yes**, is it with soap? | 1. No 2. Yes |  |  |
|  | **Question to the mother:** do you wash the hands of your under-five child after he/she eats? | 1. No 2. Yes | **If the answer is no, skip to question # 148** | |
|  | **If the answer to the question # 198 is yes**, is it with soap? | 1. No 2. Yes |  |  |

**4.5. Observation checklist**

**To the interviewer: Don’t ask anyone from the household members, simply observe and encircle your answer!**

| **No.** | **Questions** | **Options** | **Skip** | **Remark** |
| --- | --- | --- | --- | --- |
|  | Does the household have watch? | 1. No 2. Yes |  |  |
|  | Does the household have a television? | 1. No 2. Yes |  |  |
|  | Does the household have a radio? | 1. No 2. Yes |  |  |
|  | Is the house floor made of concrete? | 1. No 2. Yes |  |  |
|  | Does the household have separate kitchen? | 1. No 2. Yes |  |  |
|  | Does the household have a separate room for domestic animals? | 1. No 2. Yes |  |  |
|  | Does the household have a bank saving account? | 1. No 2. Yes |  |  |
|  | Does the household have a water storage tank? | 1. No 2. Yes |  |  |

**4.6. Spreadsheet of follow up survey for diarrheal diseases of under-five children in the Handwashing arm**

***To the interviewer: please encircle as well as write the answer!***

| **Name of the under-five child** | **Two Bars of soap will be given to the household once every two weeks for four months** | | | **Soap Wrappers will be collected from each households** | | | **Incidence of diarrheal diseases in the following series of two weeks** | | | | |
| --- | --- | --- | --- | --- | --- | --- | --- | --- | --- | --- | --- |
|  | **Series of two weeks** | **Did you wash your hands with soap and water?** | | **Series of two weeks** | **Do you collect the soap wrappers?** | | **Series of two weeks** | **Did the child have diarrhea in the past series of two weeks?** | | **If yes, for how long the diarrhea stayed?** | |
|  | **At the beginning of the:** |  |  | **In the:** |  |  | **At the end of the:** |  |  |  |  |
|  |  |  |  |  |  |  |  |  |  | **Date when started** | **Date when ended** |
|  | 1^st^ two weeks | Yes | No | 1^st^ two weeks | Yes | No | 1^st^ two weeks | Yes | No |  |  |
|  | 2^nd^ two weeks | Yes | No | 2^nd^ two weeks | Yes | No | 2^nd^ two weeks | Yes | No |  |  |
|  | 3^rd^ two weeks | Yes | No | 3^rd^ two weeks | Yes | No | 3^rd^ two weeks | Yes | No |  |  |
|  | 4^th^ two weeks | Yes | No | 4^th^ two weeks | Yes | No | 4^th^ two weeks | Yes | No |  |  |
|  | 5^th^ two weeks | Yes | No | 5^th^ two weeks | Yes | No | 5^th^ two weeks | Yes | No |  |  |
|  | 6^th^ two weeks | Yes | No | 6^th^ two weeks | Yes | No | 6^th^ two weeks | Yes | No |  |  |
|  | 7^th^ two weeks | Yes | No | 7^th^ two weeks | Yes | No | 7^th^ two weeks | Yes | No |  |  |
|  | 8^th^ two weeks | Yes | No | 8^th^ two weeks | Yes | No | 8^th^ two weeks | Yes | No |  |  |

**4.7. Spreadsheet of follow up survey for diarrheal diseases of under-five children in the control arm**

***For the interviewer, please encircle as well as write the answer!***

| **Name of the under-five child** | **Incidence of diarrheal diseases in the following series of two weeks** | | | | |
| --- | --- | --- | --- | --- | --- |
|  | **Series of two weeks** | **Did the child have diarrhea in the past series of two weeks?** | | **If yes, for how long the diarrhea stayed?** | |
|  | **At the end of the:** |  |  | **Date when the diarrhea started** | **Date when the diarrhea ended** |
|  | 1^st^ two weeks | Yes | No |  |  |
|  | 2^nd^ two weeks | Yes | No |  |  |
|  | 3^rd^ two weeks | Yes | No |  |  |
|  | 4^th^ two weeks | Yes | No |  |  |
|  | 5^th^ two weeks | Yes | No |  |  |
|  | 6^th^ two weeks | Yes | No |  |  |
|  | 7^th^ two weeks | Yes | No |  |  |
|  | 8^th^ two weeks | Yes | No |  |  |
